# Effect of patient-specific scapular morphology on the glenohumeral joint force and shoulder muscle force equilibrium: a study of rotator cuff tear and osteoarthritis patients

**DOI:** 10.3389/fbioe.2024.1355723

**Published:** 2024-05-14

**Authors:** Alexandra Oswald, Johanna Menze, Hanspeter Hess, Matthijs Jacxsens, J. Tomas Rojas, Alexandre Lädermann, Michael Schär, Stephen J. Ferguson, Matthias A. Zumstein, Kate Gerber

**Affiliations:** ^1^ School of Biomedical and Precision Engineering, Personalized Medicine Research, University of Bern, Bern, Switzerland; ^2^ Department of Orthopedic Surgery and Traumatology, Kantonsspital St Gallen, St. Gallen, Switzerland; ^3^ Department of Orthopedic Surgery, Clinica Santa Maria, Providencia, Chile; ^4^ Division of Orthopaedics and Trauma Surgery, Hôpital de La Tour, Meyrin, Switzerland; ^5^ Division of Orthopaedics and Trauma Surgery, Department of Surgery, Geneva University Hospitals, Geneva, Switzerland; ^6^ Faculty of Medicine, University of Geneva, Geneva, Switzerland; ^7^ FORE (Foundation for Research and Teaching in Orthopedics, Sports Medicine, Trauma, and Imaging in the Musculoskeletal System), Meyrin, Switzerland; ^8^ Department of Orthopaedic Surgery, Inselspital, Bern, Switzerland; ^9^ Institute for Biomechanics, ETH Zurich, Zürich, Switzerland; ^10^ Shoulder, Elbow and Orthopaedic Sports Medicine, Orthopaedics Sonnenhof, Bern, Switzerland

**Keywords:** glenohumeral joint, osteoarthritis, rotator cuff tear, joint biomechanics, patient-specific simulations, musculoskeletal modeling

## Abstract

**Introduction:** Osteoarthritis (OA) and rotator cuff tear (RCT) pathologies have distinct scapular morphologies that impact disease progression. Previous studies examined the correlation between scapular morphology and glenohumeral joint biomechanics through critical shoulder angle (CSA) variations. In abduction, higher CSAs, common in RCT patients, increase vertical shear force and rotator cuff activation, while lower CSAs, common in OA patients, are associated with higher compressive force. However, the impact of the complete patient-specific scapular morphology remains unexplored due to challenges in establishing personalized models.

**Methods:** CT data of 48 OA patients and 55 RCT patients were collected. An automated pipeline customized the AnyBody™ model with patient-specific scapular morphology and glenohumeral joint geometry. Biomechanical simulations calculated glenohumeral joint forces and instability ratios (shear-to-compressive forces). Moment arms and torques of rotator cuff and deltoid muscles were analyzed for each patient-specific geometry.

**Results and discussion:** This study confirms the increased instability ratio on the glenohumeral joint in RCT patients during abduction (mean maximum is 32.80% higher than that in OA), while OA patients exhibit a higher vertical instability ratio in flexion (mean maximum is 24.53% higher than that in RCT) due to the increased inferior vertical shear force. This study further shows lower total joint force in OA patients than that in RCT patients (mean maximum total force for the RCT group is 11.86% greater than that for the OA group), attributed to mechanically advantageous muscle moment arms. The findings highlight the significant impact of the glenohumeral joint center positioning on muscle moment arms and the total force generated. We propose that the RCT pathomechanism is related to force magnitude, while the OA pathomechanism is associated with the shear-to-compressive loading ratio. Overall, this research contributes to the understanding of the impact of the complete 3D scapular morphology of the individual on shoulder biomechanics.

## 1 Introduction

Common degenerative pathologies of the glenohumeral joint include rotator cuff tears (RCTs), the most common source of shoulder disability (Cadogan et al., 2011; Yamaguchi et al., 2006), and osteoarthritis (OA) of the glenohumeral joint, which has been found in 5%–17% of patients presenting with joint pain (Ibounig et al., 2021). Although the causes of these diseases are undoubtedly multifactorial, previous studies have identified distinct scapular morphological metrics that differentiate the pathological groups. Two such metrics, defined on true anteroposterior (AP) radiographs, are the critical shoulder angle (CSA), which characterizes the relative position of the glenoid process and the acromion, and glenoid inclination (GI) (Moor et al., 2013). It has been shown that OA patients are more likely to have a CSA smaller than 30° (Moor et al., 2013), while RCT patients typically have a CSA greater than 35° and a more superiorly inclined glenoid (Moor et al., 2016; Nyffeler and Meyer, 2017). Studies have reported a positive association between the CSA and vertical shear on the glenohumeral joint and, hence, joint instability and rotator cuff recruitment (Moor et al., 2016; Villatte et al., 2020; Viehöfer et al., 2016). It has been suggested that this could lead to joint overloading and increased muscle degeneration, postulating a relationship to the development of the RCT pathology. Villatte et al. (2020) further showed that a reduced CSA increased the joint compressive force, which they suggested could contribute to the joint wear patterns of OA patients. Although these parameter studies demonstrated the effect of specific aspects of the scapula morphology on shoulder biomechanics, the full complex three-dimensional (3D) scapular morphology and the interdependence of specific morphological aspects in RCT and OA patients remain unstudied. Statistical shape modeling of the scapula has shown that within the principal modes of variation of the scapula, the morphology of the coracoid, acromion, and glenoid processes is not independent of one another (Jacxsens et al., 2020). Therefore, to provide further insight into the morphology–biomechanical relationships of RCT and OA patients, patient-specific models considering full scapula geometry are necessary. The computational study of patient glenohumeral biomechanics could provide an insight into different pathological groups; however, large-scale studies have never been performed, most likely due to the effort needed to set up patient-specific shoulder models.

To enable large-scale patient-specific analysis, we developed an automated pipeline to efficiently create tailored biomechanical models for large quantities of patient data based on computed tomography (CT) images. Using this pipeline, we present a study to investigate how the patient-specific geometry of the glenohumeral joint, particularly in patients with RCT and OA, influences the moment arms of the rotator cuff muscles and consequently affects the muscle activation and generated forces.

## 2 Methods

### 2.1 Data collection

Following approval from the Cantonal Ethics Committee of Bern, Switzerland (KEK-N. 2016-01858), retrospective CT data (in a plane resolution of [0.42–0.99] mm in the sagittal and coronal planes and a slice thickness of [0.30–0.90] mm) on the affected shoulder of 48 primary OA patients (mean age: 59 ±8 years; 17 left and 31 right; 28 males and 20 females; clinically screened for an intact rotator cuff) and 55 posterosuperior RCT patients (mean age: 57 ±9 years; 15 left and 40 right; 28 males and 27 females) acquired during normal clinical routine between 2010 and 2018 at the Inselspital, University Hospital of Bern were collected for this study. The CSA, defined by Moor et al. (2013) as the angle between the line connecting the superior and inferior margins of the glenoid and a line connecting the inferior glenoid margin and the most lateral boarder of the acromion, was manually measured from each CT as a two-dimensional projection on the scapular plane [defined by Suter et al. (2015) between the center of the glenoid, the apex of the inferior scapular angle, and the point at the medial border intersecting with the scapular spine]. The scapula and humerus were manually segmented by clinical experts from each CT, resulting in three-dimensional surface meshes (JR and MJ, both fellowship-trained). Eleven landmarks representative of the scapular morphology were picked by the same experts on the resulting mesh (the most lateral point on the coracoid process, the most anterior–lateral point on the acromion process, the most posterior–lateral point on the acromion process, the angulus inferior and superior, and the most inferior, superior, lateral, and medial points from the glenoid rim) (Mimics 10.1, Materialise, Leuven, Belgium).

### 2.2 Patient-specific modeling pipeline

**FIGURE 1:**
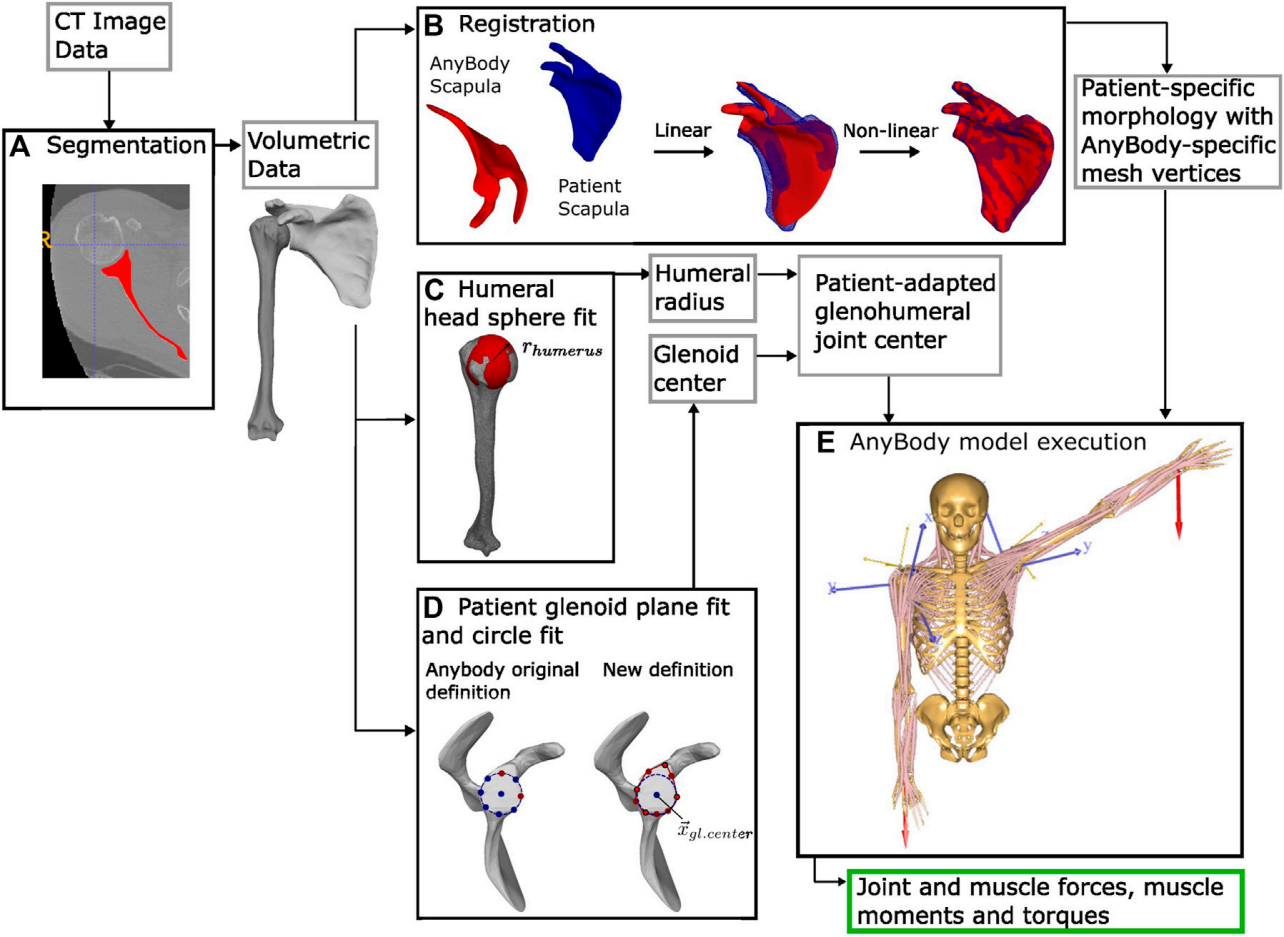
Data flow processing beginning from the patient’s CT image data. **(A)** Segmentation. **(B)** Registration of the AnyBody™ scapula to the patient scapula. **(C)** Sphere fit of the medial humeral head to approximate the radius of the glenohumeral joint. **(D)** Left: original circle fit to the glenoid rim from anterior and inferior glenoid points. Right: our definition, with the plane of the glenoid fit to nine points around the glenoid rim. Projected on this plane, a circle fit to the inferior five points defines the glenoid center. Using **(C,D)**, the patient-adapted glenohumeral joint is defined. **(E)** Inverse kinematic modeling using the AnyBody™ modeling system: the muscle forces, moments, and torques, as well as the joint force result.

An automated patient-specific modeling pipeline was implemented for the AnyBody™ modeling environment [AnyBody™ Modeling System ver. 7.3.4, AnyBody Technology A/S, Aalborg, Denmark (Damsgaard et al., 2006)] for the incorporation of the patient-specific scapular morphology and glenohumeral joint geometry. The pipeline consisted of an automatic morphing of the AnyBody™ reference scapula to the patient-specific scapula, followed by a customization of the glenohumeral joint to reflect the patient-specific glenoid inclination and glenoid cavity shape and the patients’ humeral head diameter (Figure 1). All algorithms including those used for verification were developed using Python.

#### 2.2.1 Generic musculoskeletal modeling

The AnyBody™ shoulder model is defined by the connections of the humerus, scapula, and clavicle, based on anthropometric data and modeling assumptions from the Dutch shoulder model (van der Helm, 1994; Van der Helm et al., 1992). The glenohumeral joint enables three rotational degrees of freedom (DOFs) but no translation. Spherical joints additionally link the clavicle to the sternum (sternoclavicular joint), and the acromion process with the clavicula (acromioclavicular joint). The distance between the coracoid process and the clavicula is constrained by a fixed distance representing the conoid ligament. Moreover, the scapula articulates with the rib cage using the generic shoulder rhythm of AnyBody™, which couples scapular motion to humeral elevation (de Groot and Brand, 2001). The shoulder joint is spanned by 16 muscles, separated into 118 discrete bundles, representing the entirety of the origin and insertion sites of each muscle. Some muscles, including the deltoid, have surfaces over which they are constrained to wrap (cylinder, spheres, or ellipsoids). The strength of each muscle is directly proportional to its physiological cross-sectional area and assumed to be independent of the muscle length during motion (Andersen et al., 2021). AnyBody™ uses an inverse dynamic approach to calculate the required muscle forces and resultant joint reaction forces for a given kinematic input. This overdetermined system, for which multiple muscles are responsible for generating force about a single DOF, is solved using a third-order polynomial cost function to determine the optimal recruitment based on the tradeoff between muscle synergy, muscle force distribution, and physiological muscle activation times (Andersen et al., 2021).

#### 2.2.2 Scapular anatomy morphing

To morph the generic AnyBody™ model scapula to the patient’s bone morphology, a two-step algorithm for non-rigid registration was implemented (Figure 1B). First, an affine alignment using the iterative closest point (ICP) algorithm (Besl and McKay, 1992; Chen and Medioni, 1992) with coarse initialization of surface models using the principal axis and centroid of each surface was applied to the full scapular surface meshes (sampled at 5,000 points). Non-linear morphing was thereafter performed with large deformation diffeomorphic metric mapping (LDDMM) [deterministic atlas algorithm, Deformetrica (Durrleman et al., 2014)]. To account for high variations in the glenoid process geometry, four points on the glenoid (the most superior, anterior, inferior, and posterior aspects of the glenoid rim) were constrained during LDDMM optimization. During registration, AnyBody™ defines the muscle insertion points and muscle wrapping functions of the model based on specific points on the morphed bone mesh. Thus, through scapular morphing, the locations of the origins of the rotator cuff muscles, the deltoid, biceps brachialis, coracobrachialis, triceps, and teres major as well as the insertion sites of the trapezius, pectoralis minor, levator scapulae, serratus anterior, and rhomboideus muscles were automatically updated to the patient specific anatomy. The accuracy of the non-rigid registration was verified on all the registered scapulae (*N* = 103) as the error (the Hausdorff distance and the mean point-to-surface distance) compared to the original segmented patient scapulae. The accuracy was additionally verified at the 11 user-defined landmarks as the point-to-point distance. This initialization of the model based on the generic standing posture of the shoulder model was chosen over the static supine CT position of the scapula due to the substantial discrepancies in scapular alignment between the supine and standing positions (Matsumura et al., 2020).

#### 2.2.3 Model scaling

The remaining model components, which include the humerus, the rest of the human skeleton, and the muscle length defined by the muscle insertion points (excluding the muscles listed above), muscle volumes, and wrapping surfaces, were uniformly scaled based on the height of the patient’s glenoid, $gh_{patient}$ , according to a correlation determined by Piponov et al. (2016) of an increase of 7.4 cm in the height per 1 mm of the glenoid height increase. The patient-specific scapula did not undergo any additional scaling. Additionally, they showed that the glenoid is, on average, 2.9 mm shorter in females than in males (Piponov et al., 2016). As the generic AnyBody™ model, ${\text{gh}}_{Anybody}$ , represents a male patient, 2.9 mm was subtracted from the glenoid height when scaling for female patients. The formula for the patient height in mm, $h_{\text{patient}}$ , was thus (Eq. 1):

$$h_{patient}=1800+\left({\text{gh}}_{patient}-{\text{gh}}_{Anybody}-2.9\cdot SEX\right)\cdot 74,$$ (1)

where SEX=1 for females and SEX=0 for males. Weight was scaled linearly based on the patient height.

#### 2.2.4 Patient-adapted glenohumeral joint model

To account for high variation in the patient-specific glenoid process, the glenohumeral joint definition was adapted from the AnyBody™ Model Repository (Lund et al., 2022). In the generic model, the glenohumeral joint center is defined as the apex of a cone, with a base defined as a circle fit to the superior and anterior points of the glenoid cavity (Figure 1D, left). For stable motion, the total sum of the forces acting on the glenohumeral joint must be contained within this cone. This definition does not consider varying glenoid inclinations. Alternatively, we defined the joint center, ${\vec{x}}_{gh.joint}$ , relative to the center of the glenoid cavity, ${\vec{x}}_{gl.\;center}$ , according to the following (Equation 2):

$${\vec{x}}_{gh.joint}={\vec{x}}_{gl.\;center}+\vec{n}\cdot r_{humerus},$$ (2)

where ${\vec{x}}_{gl.\;center}$ is the center of a circle fit to the inferior two-thirds of the glenoid (De Wilde et al., 2004), projected onto a plane with normal $\vec{n}$ fit to nine points distributed around the glenoid rim (Figure 1D). The humeral $r_{humerus}$ was defined by using a best-fit sphere, approximated as the sphere minimizing the sum of the squared residuals of the surface of the anatomical head of the humerus. An additional 2 mm was added to account for the conservative uniform cartilage coverage [mean thickness of the humeral cartilage reported to be from 0.89 to 1.74 mm (Fox et al., 2008; Soslowsky et al., 1992; Zumstein et al., 2014), and mean cartilage thickness of the glenoid reported at approximately 2 mm (Zumstein et al., 2014; Loy et al., 2018) in healthy patient cohorts]. This adjustment in the glenohumeral joint position reorients the stability constraint cone to align with the patient-specific orientation of the glenoid process and scales the cone according to the glenoid rim and radius of the patient-specific humerus.

### 2.3 Biomechanical simulation

For each patient-specific model in AnyBody™, the total glenohumeral joint forces and the compression, vertical shear, and horizontal shear components were calculated in 12 increments, over a 0°–120° motion arc (10N weighted abduction in the frontal plane and weighted flexion in the sagittal plane). To compare the two patient groups, the forces of each patient were normalized by their body weight. Following the simulation, the results from the left-side scapulae were mirrored about the sagittal plane and compiled with the right-side results. For each patient group, the mean and standard deviation of the forces were calculated. The muscle torque, τ , of each of the rotator cuff muscles and of the deltoid was calculated from the muscle moment arm, r , and force, F , output from the model, using the formula τ=r×F (Tipler and Mosca, 2015). Statistical significance was evaluated using statistical parametric mapping (SPM) (Penny et al., 2011) (two-tailed, two-sample t-test with an alpha value of 0.05 evaluated for each measurement point).

#### 2.3.1 Instability ratio

**FIGURE 2:**
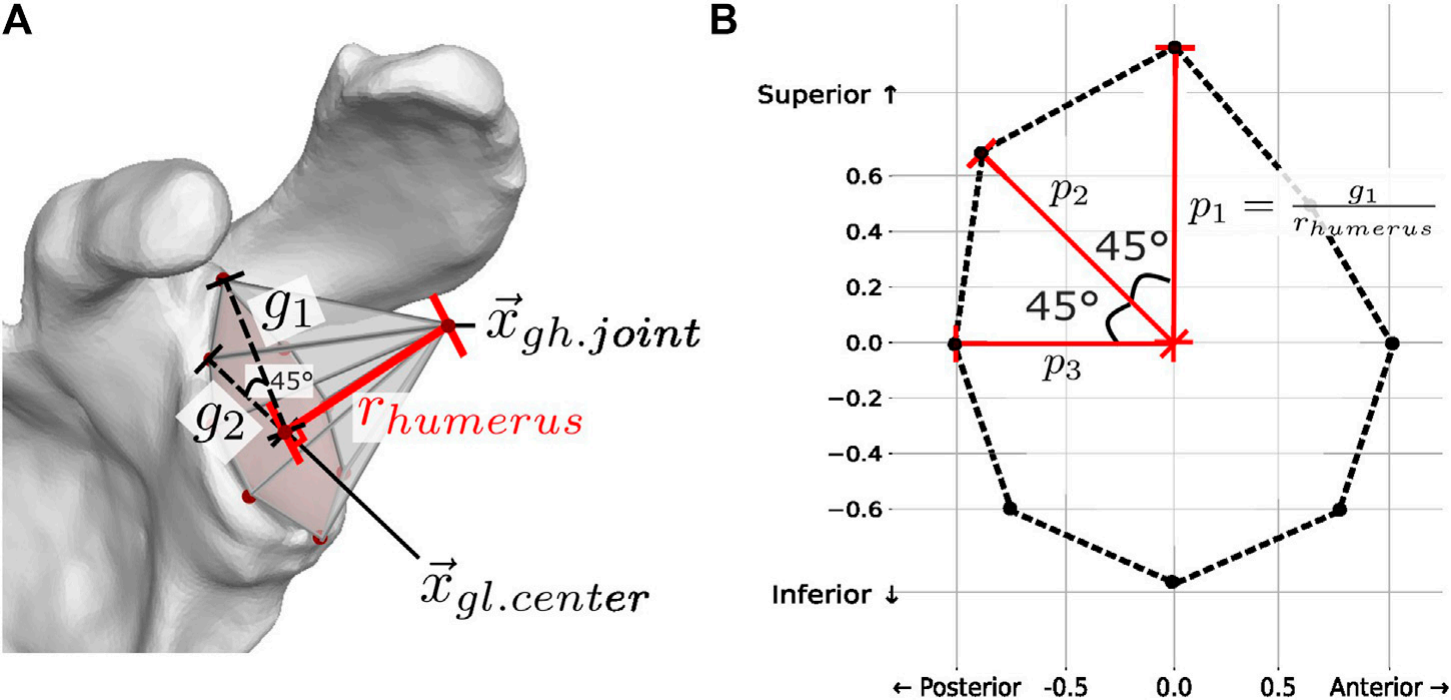
**(A)** Passive stability $p_{n}$ is defined at *n* = 8 points at 45° increments as the ratio between the glenoid radius $g_{n}$ and the humeral radius $r_{humerus}$ (constant for all 8 points): $p_{n}=\frac{g_{n}}{r_{humerus}}$ . **(B)** Plot of the resulting passive stability polygon.

The efficacy of concavity compression in stabilizing the glenohumeral joint has been assessed through the stability ratio, which measures the maximum translational force stabilized in a specific direction divided by the applied compressive force, particularly in cadaveric models (Halder et al., 2001; Lazarus et al., 1996). In biomechanical modeling, the vertical $\rho_{vert}$ and horizontal $\rho_{horz}$ instability ratios were employed to characterize the ratio of vertical and horizontal shears to the compression force relative to the motion arc (Moor et al., 2016; Gerber et al., 2014). For our model, the instability ratios were plotted radially, with the magnitude defined as $r_{ins}=\sqrt{\rho_{vert}^{2}+\rho_{horz}^{2}}$ with the angle in the glenoid plane $\theta =\arctan\left(\rho_{vert}/\rho_{horz}\right)$ superimposed on the radial passive stability at *n* = 8 points at 45 increments around the glenoid cavity. Passive stability, $p_{n\;}$ , was defined as the ratio of the glenoid radius, $g_{n}$ , to the humeral radius, rhumerus (Eq. 3):

$$p_{n}=\frac{g_{n}}{r_{humerus}},$$ (3)

where the radius of the glenoid $g_{1-8}$ was defined relative to the center of the glenoid cavity ( $\left.{\vec{x}}_{gl.\;center}\right)$ (Figure 2).

## 3 Results

The mean measured CSA was $32.73\pm 4.89^{\circ }$ for the RCT patients and $24.88\pm 6.15^{\circ }$ for the OA patients. The mean GI was 81.42° ± 5.89° for the RCT patients and 86.13 ± 7.16 for the OA patients. Both the CSA and GI were significantly different between the two patient groups (*p* < 0.001 in both cases).

### 3.1 Bone registration accuracy

The mean and standard deviation of the point-to-surface distance and the Hausdorff distance for the non-rigid registration of the scapulae (*N* = 103) were 0.34 ±0.08 mm and 2.29 ±0.9 5 mm, respectively. The point-to-point accuracies of the 11 defined landmarks are given in Table 1.

**TABLE 1:** Group mean and standard deviation (STD) of the Hausdorff distance, mean, and median point-to-surface distance.

	Mean of all patients [mm]	STD of all patients [mm]
Hausdorff distance	2.295	0.954
Mean point-to-surface distance	0.338	0.076
Median point-to-surface distance	0.262	0.043
Mean point-to-surface distance at landmarks [mm]		
Lateral coracoid process	0.563	0.672
Anterior lateral acromion process	0.552	0.508
Posterior lateral acromion process	0.564	0.396
Angulus inferior	0.629	0.418
Angulus superior	0.837	0.510
Inferior glenoid rim	0.398	0.278
Anterior glenoid rim	0.361	0.238
Posterior glenoid rim	0.348	0.276
Superior glenoid rim	0.450	0.305

The point-to-surface landmark distances are presented for the coracoid, glenoid, and acromion processes, as well as for the angulus superior and inferior of the scapula.

### 3.2 Glenohumeral joint reaction forces

**FIGURE 3:**
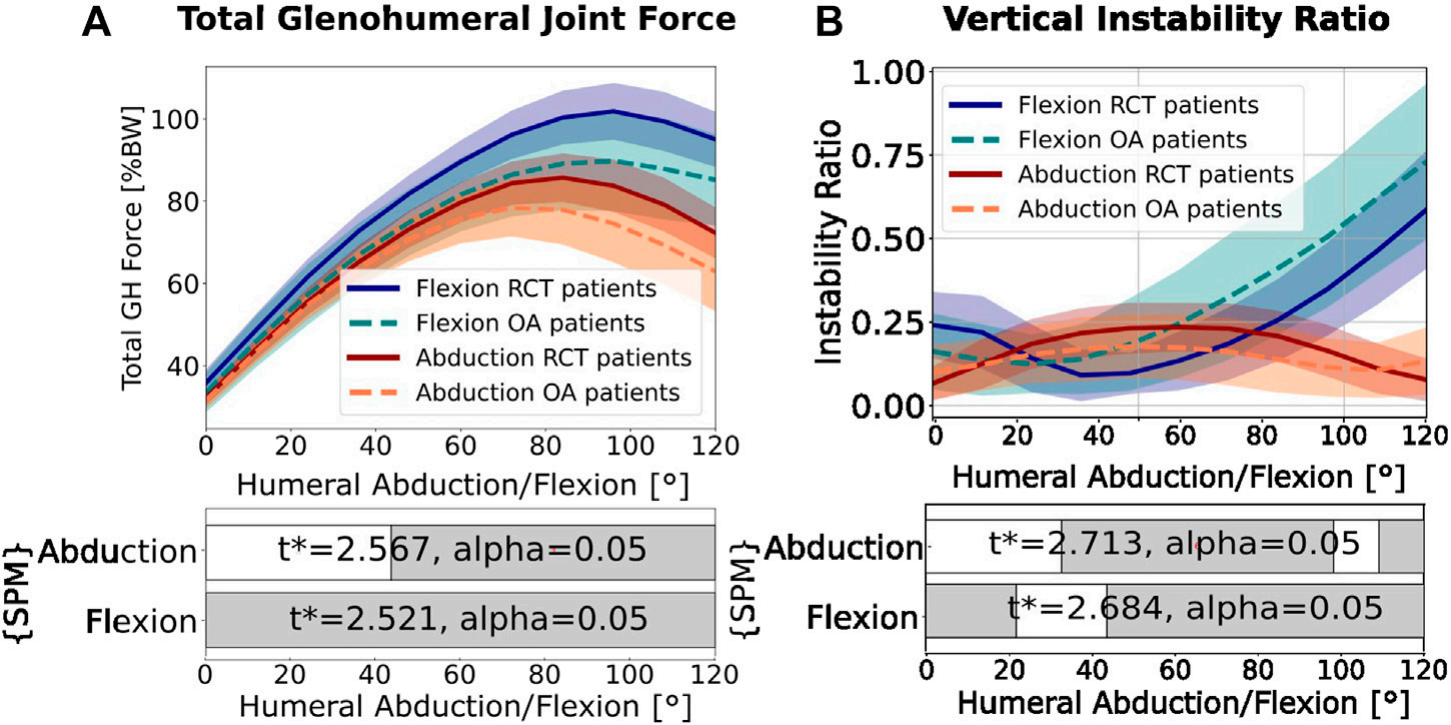
**(A)** Total force magnitude. **(B)** Vertical instability ratio (ratio of vertical shear to compressive forces). In both graphs, red indicates abduction of 0°–120° and blue indicates flexion of 0°–120°. The mean values from the osteoarthritis (OA) patients are represented by dotted lines. The mean values from the rotator cuff tear (RCT) patients are represented by full lines. The colored area represents the standard deviation. The statistical parametric mapping (SPM) bars indicate statistically significant differences between the RCT and OA groups from a two-tailed, two-sample t-test with an annotated $t^{*}$ value, and α=0.05 . Gray indicates statistical significance: *p* < 0.050.

In abduction, the mean total force was significantly higher in the RCT group (*p <* 0.050 for 48°–120° of abduction), and the mean magnitude of the maximum total force vector for the RCT group (85.73 ± 5.90 %BW) was 8.56% greater than that for the OA group (78.39 ± 6.98 %BW) (Figure 3A). In flexion, the total force was significantly higher for the RCT group over the entire flexion arc (*p <* 0.050 for 0°–120*°* of flexion) with the mean maximum total force for the RCT group (101.84 ± 6.89 %BW) and 11.86% greater than that of the OA group (89.76 ± 12.21 %BW) (Figure 3B).

**FIGURE 4:**
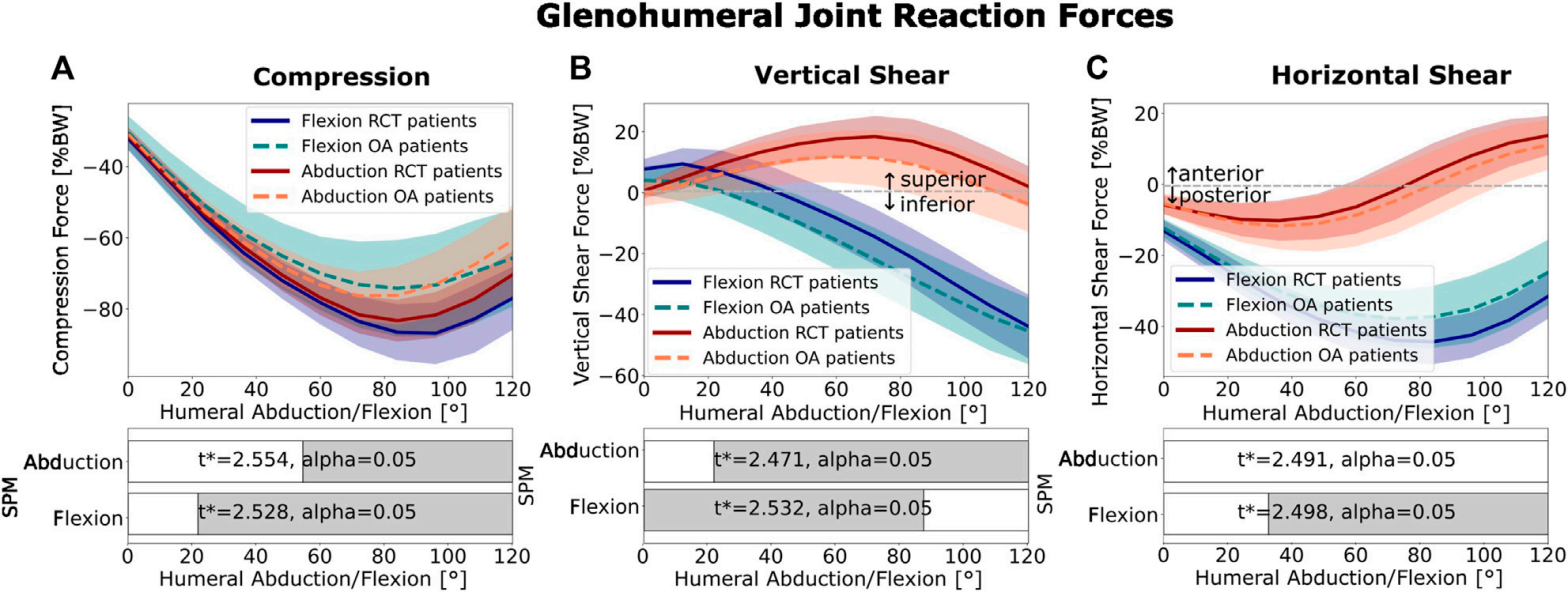
Glenohumeral joint reaction forces: **(A)** compression, **(B)** vertical shear, and **(C)** horizontal shear. Abduction is shown in red and flexion in blue. The mean RCT patient forces are shown as dark solid lines. The mean OA patient group is represented by light dotted lines. Colored areas show the standard deviation. The SPM bars below each graph indicate statistically significant differences between the RCT and OA groups from a two-tailed, two-sample t-test with an annotated $t^{*}$ value, and α=0.05 . Gray indicates statistical significance: *p* < 0.050.

The RCT patients consistently demonstrated a higher mean glenohumeral joint compression force (*p <* 0.050 between 25° and 120° of abduction and 56°–120° of flexion; Figure 4A). In abduction, the RCT patients demonstrated significantly higher shear force magnitudes in the superior direction (*p <* 0.050 for 24°–120° of abduction), whereas in flexion, the OA patients had significantly higher shear force magnitudes (*p <* 0.050 for 0°–88° of flexion) but in the inferior direction (Figure 4B). For both patient groups, in abduction, the vertical shear force increased in magnitude until 72°, followed by a symmetric decrease. In flexion, the vertical shear force profile showed a constant increase in the inferior vertical shear force after 10° motion for both patient groups. The horizontal shear in abduction was not significantly different between the two patient groups, and the force direction remained centered (Figure 4C, red). In flexion, however, in both groups, the horizontal shear force tended to be posterior and was higher in the magnitude in the RCT group (*p <* 0.050 for 36°–120° of flexion; Figure 4C, blue).

### 3.3 Instability ratios

The vertical instability ratio (the ratio of vertical shear to compression force) in abduction was significantly higher in RCT patients than that in OA patients (*p <* 0.050 for 30°–100° of abduction; Figure 3B), with a maximum of 32.80% higher (23.44% at 60° of abduction) than for the OA group (17.65% at 48° of abduction). In flexion, the instability ratio increased continuously in both patient groups but was 24.53% higher (maximum) for the OA group (maximum of 72.98% at 120° of flexion) than that for the RCT group (maximum of 58.6% at 120° of flexion). The horizontal instability ratio (ratio of horizontal shear to compressive forces) was similar in both motion types for both patient groups.

**FIGURE 5:**
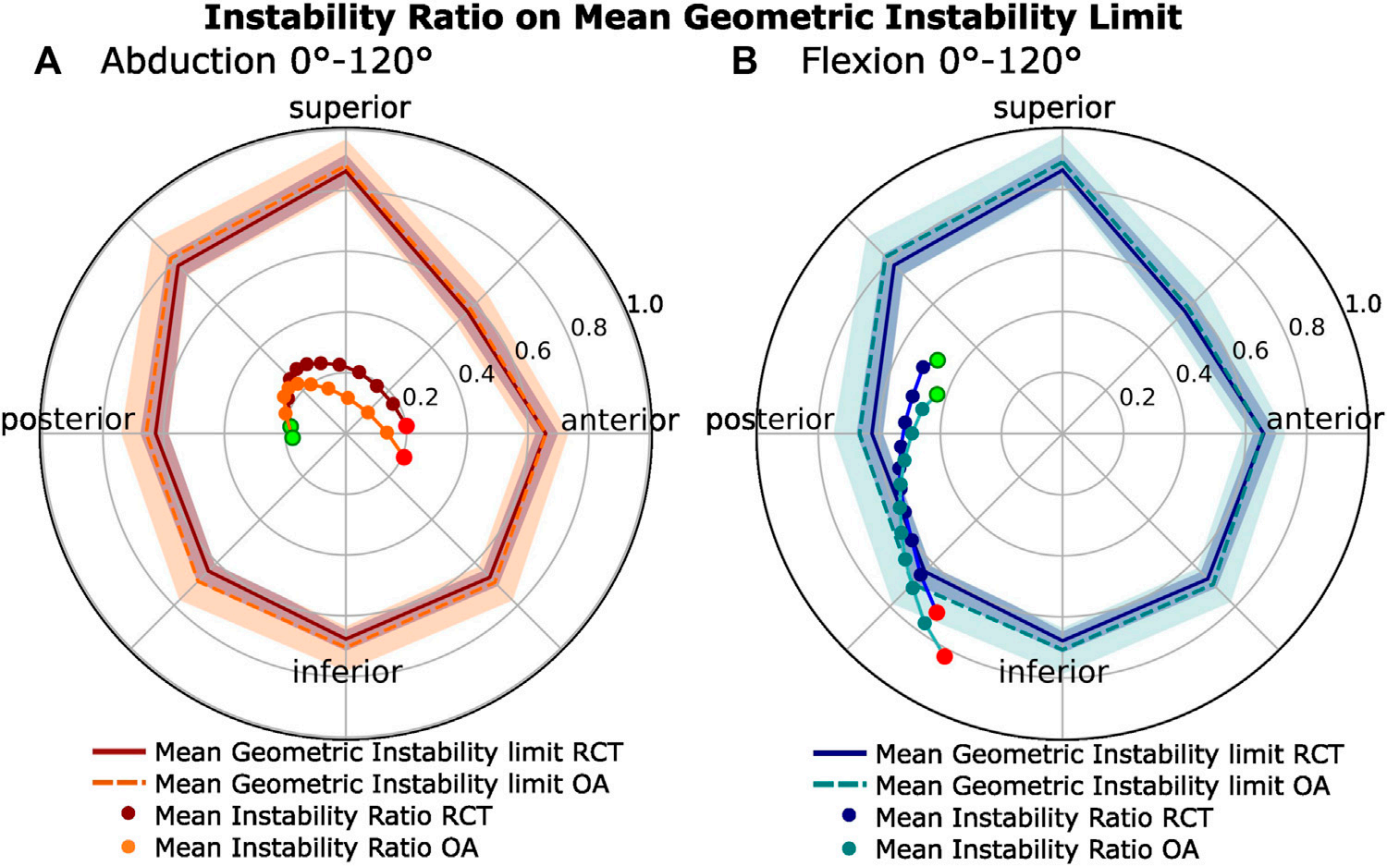
Passive glenohumeral stability limits compared to the dynamic instability ratios. **(A)** Abduction. **(B)** Flexion. Mean polygons representing the passive glenohumeral stability limit (see Section 2.3.1) for the OA (light) and RCT (dark) patient groups with standard deviation. Within each polygon, the radial plot of the horizontal instability ratio (horizontal shear over compression) and vertical instability ratio (vertical shear over compression) for both patient groups over 0°–120° of motion are shown. The starting point (0°) is shown in green, while the end point (120°) is shown in red.

The mean area of the passive-stability polygon of the OA group was 6.67% larger for the OA group than that for the RCT group (*p =* 0.008) (Figure 5, solid and dashed polygon contour). To illustrate the variance in the passive stability within pathological groups, we calculated the difference in the area of the polygons plus or minus one standard deviation from the mean (shaded polygons in Figure 5). The area from the OA patients was found to be 49.25% greater than that of the RCT patients. Over abduction (Figure 5A), there was a constant anterior shift in the orientation of the instability ratio over the motion arc in both groups. In abduction (Figure 5A; Section 2.3.1), the maximum magnitude of the instability ratio was 0.267 for the RCT patients and 0.241 for the OA patients at 36° of humeral abduction, resulting in a maximum shear-to-compression force ratio in a posterior–superior direction in the glenoid plane. For both patient groups, these values were well within the passive stability limit (Figure 5A). In flexion (Figure 5B), the instability ratio was oriented more inferiorly and posteriorly than that in abduction for both patient groups. The maximum mean magnitude of the instability ratio was 0.716 for the RCT patients and 0.825 for the OA patients at 120° of humeral flexion, resulting in a maximum shear-to-compression force ratio in a posterior–inferior direction in the glenoid plane. The steady increase in shear-to-compressive force magnitude over the whole flexion arc leads to a dynamic instability ratio exceeding the mean passive limit in both patient groups, particularly in the OA patient group, from 75° to 120°.

### 3.4 Muscle torque and moment arm

**FIGURE 6:**
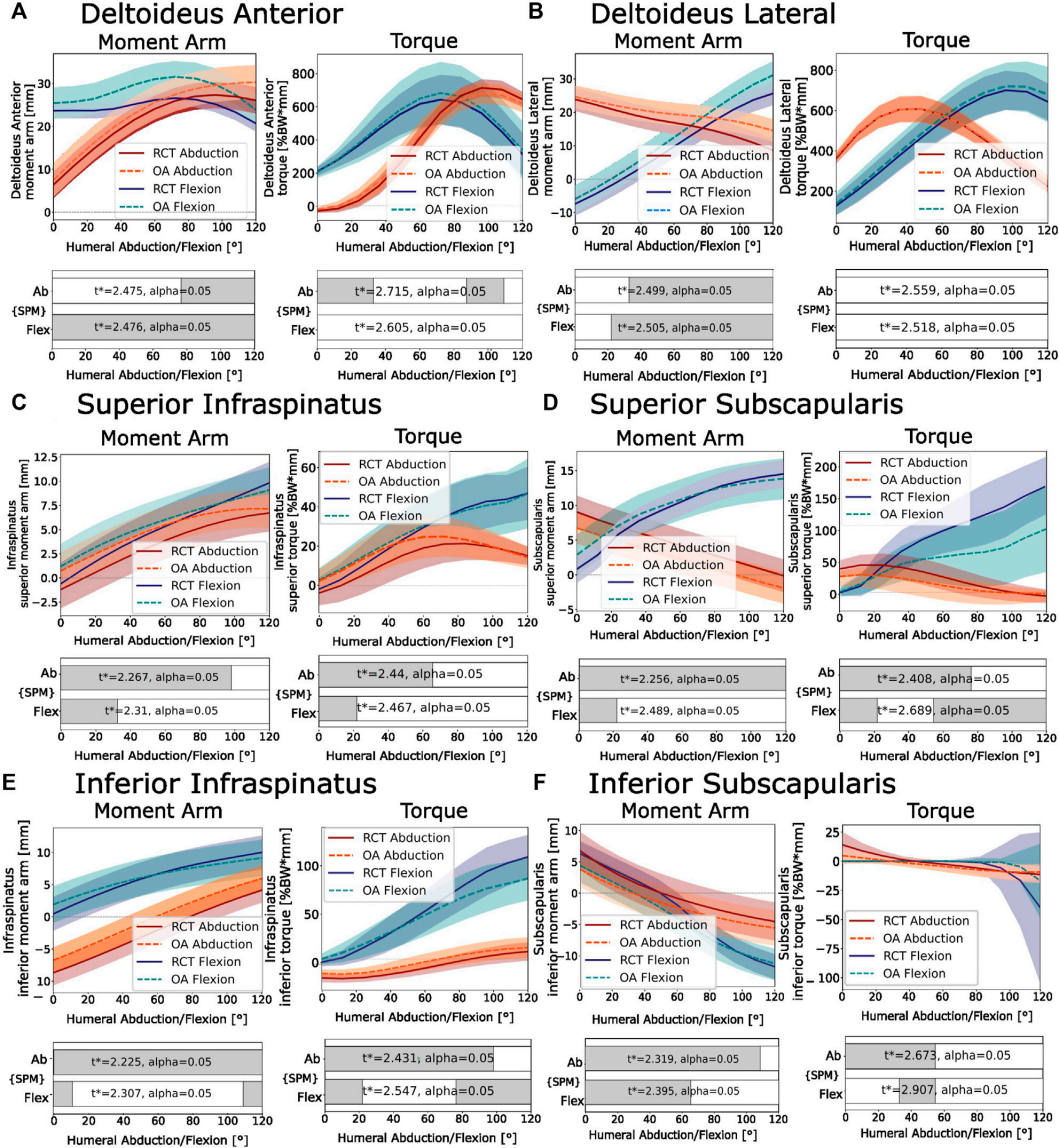
Moment arm (left) and torque (right). **(A)** Anterior deltoid. **(B)** ateral deltoid. **(C)** Superior infraspinatus. **(D)** Superior subscapularis. **(E)** Inferior infraspinatus. **(F)** Inferior subscapularis. Negative moment arms facilitate antagonistic motion from that muscle. Negative torque works against the primary movers. Abduction of 0°–120° is shown in red, and flexion of 0°–120° is shown in blue. RCT patients are shown as dark solid lines. OA patients are shown as light dotted lines. The SPM bars below each figure indicate statistically significant differences between the RCT and OA groups from a two-tailed, two-sample t-test with an annotated $t^{*}$ value, and α=0.05 . Gray indicates statistical significance: *p* < 0.050.

In abduction, the moment arm of the subscapularis decreased steadily over the motion arc, switching to facilitate abduction at higher humeral abduction angles (Figures 6D, F, red, left). Over the agonistic portion of the motion arc, the moment arm of the RCT patients had a significant mechanical advantage over that of the OA patients (*p* < 0.050 for 0°–120° in superior muscle and for 0°–110° in inferior muscle). In humeral flexion, the superior subscapularis acted purely as an agonist, with an increasing moment arm at higher humeral flexion angles, with a similar result in both the OA and RCT groups (Figure 6D, blue, left). In both flexion and abduction, the inferior and superior infraspinatus muscle showed an increasingly agonistic moment arm (Figures 6C, E, left), with the OA group having a significantly longer moment arm than the RCT group (*p* < 0.050 for 0°–95° in abduction; for 0°–35° in flexion for the superior muscle part; and for 0°–120° in abduction for the inferior muscle part) with the exception of the inferior portion of the infraspinatus in flexion, which was similar in both patient groups. The moment arm of the deltoid, as the prime mover in abduction and flexion, is presented for the lateral and anterior portions of the muscle, as shown in Figures 6A, B, left. The OA group had longer moment arms than the RCT group (*p* < 0.050 for 75°–120° in abduction; for 0°–120° in flexion for the anterior deltoid; for 35°–120° in abduction; and 22°–120° in flexion for the lateral deltoid).

Generally, the maximum torque generated in the superior subscapularis and infraspinatus in flexion was higher than that generated in abduction, with 294% and 545% higher maximum torques for the RCT group and 348% and 359% higher maximum torques for the OA group in flexion compared to abduction, respectively (Figures 6C, D, right). In abduction, the superior subscapularis torque followed an inverse-sigmoid-shaped curve, with the muscle first contributing to humeral abduction, followed by a weak contrasting abduction torque (Figure 6D, right, red), with the RCT group showing a significantly higher torque than the OA group (*p* < 0.050 for 0°–72° of abduction). In flexion, the agonist torque of the superior subscapularis increased consistently over the entire flexion arc (Figure 6D, right, blue), with the RCT group also showing significantly higher torque than the OA group (*p* < 0.050 for 48°–120° of flexion).

## 4 Discussion

Despite reported morphological differences between the RCT and OA patient groups, so far, analysis of individual patient morphologies and their effect on shoulder biomechanics has been limited by the challenging up-scaling of individual investigations to larger population sizes. Here, we present a pipeline for the automatic, patient-specific analysis of the glenohumeral joint, allowing for a large-scale comparison of the muscle and joint forces of different patient groups.

Consistent with the results of numerous prior studies, our findings underscore a significant difference in morphology between the OA and RCT patient groups, including a larger CSA and more superiorly oriented glenoid for the RCT patients than that for the OA patients (Moor et al., 2013; Daggett et al., 2015; Van Parys et al., 2021). Subsequently, we discuss the effect of these differences in morphology on the biomechanics of the glenohumeral joint.

In abduction, increased vertical shear forces acting on the glenohumeral joint of the RCT patient group compared to the OA patient group were observed (Figure 4B, red). This result was consistent with that obtained by Moor et al. (2016), who found higher vertical shear forces in abduction for more superiorly oriented glenoids. We also observed an increased vertical instability ratio in abduction for RCT patients compared to OA patients (Figure 3B, red). This is in agreement with the findings obtained by Viehöfer et al. (2016), who showed an increased vertical instability ratio in abduction for a scapula with a high CSA compared to a scapula with a normal CSA. In flexion, we showed that the comparative increase in inferior shear force compared to compression forces in OA patients resulted in a higher vertical instability ratio in the OA patients than that for the RCT patients (Figure 3B, blue).

In both flexion and abduction, increases in vertical shear-to-compression force ratio corresponded to higher activation of the rotator cuff. During abduction, a symmetric increase was observed in the instability ratio magnitude over the first 60°, followed by a decrease in the second half of the motion (Figure 5A), reflected by the increase in the agonist torque generated by the superior portions of the infraspinatus and subscapularis, followed by a decrease in the second half of the motion arc (Figure 6C, D, red, right). During flexion, the steady increase in the instability ratio magnitude (Figure 5B) on the glenohumeral joint was mirrored by a steady increase in the torque generated by the inferior rotator cuff muscles (apart from the inferior subscapularis; Figures 6C–E, blue, right). This is consistent with many studies showing that the rotator cuff generates stabilizing compressive forces, which work against destabilizing vertical shear forces (Moor et al., 2016; Viehöfer et al., 2016; Lippitt and Matsen, 1993; Wuelker et al., 1998; Bigliani et al., 1996). Comparing the two patient groups, RCT patients showed an increased compression force compared to the OA patient group in all movement types (Figure 4A). As a result, the magnitude of the total force vector was also consistently higher in the RCT patient group (Figure 3A). This is in contrast to the results obtained by Villatte et al. (2020), who found higher compression forces in a model with a low CSA than that in a model with a high CSA, when varying the lateral acromion extension. The positioning of the glenohumeral joint center significantly influences the moment arms of the rotator cuff and deltoid muscles, thereby affecting the equilibrium of forces surrounding the glenohumeral joint. One possible explanation for the disparity between our findings and those of Villatte et al. may lie in the restricted impact of varying a singular morphological parameter at the joint center as opposed to the broader range of variability observed when evaluating the comprehensive scapular morphology. As we defined it, the glenohumeral joint center was placed normal to a plane fit to the inferior glenoid rim. As OA patients tend to have more inferiorly inclined glenoids, the glenohumeral joint center was more medial and inferior to that of the RCT patients. The passive-stability polygon area and variance were greater in the OA patient group than those in the RCT patients, reflecting a greater morphological variation of the OA patients’ glenoid processes, with a tendency toward a broader structure, and less distance between the glenoid surface and the glenohumeral joint center. In our model, this shift in the relative position of the humeral head to the acromion provided the deltoid a more favorable moment arm (Figures 6A, B, left) (Nyffeler and Meyer, 2017) and resulted in a reduction in the total generated force. Clinically, the medialization of the glenohumeral joint in osteoarthritic shoulders is well documented, with the progression of joint-line medialization associated with pathological glenoid retroversion and progressive bony degeneration (Lehtinen et al., 2001; Walker et al., 2018). In contrast, in the extreme case of an anatomically reversed prosthesis, the geometry of a more medial glenohumeral joint center increases the length of the lateral deltoid moment arm and decreases the total joint reaction forces (Kontaxis and Johnson, 2009; Terrier et al., 2008).

With reduced force generated by the deltoid, a reduced compression and horizontal shear was observed in the OA patients in both abduction and flexion. However, in flexion, an increase in the vertical shear magnitude was observed (Figure 4B). In our model, the more inferior position of the glenohumeral joint center in OA patients had a favorable effect on the moment arms of the superior and inferior infraspinatus (in abduction and flexion) and superior subscapularis (in flexion) (Figures 6C–E, left). As noted by Ackland and Pandy (2009), the rotator cuff muscles do not uniquely provide compressive force; due to the lines of action of the muscles, both the subscapularis and infraspinatus have destabilizing potential, defined as a muscle that provides more shear than compressive force over a certain range of motion. The subscapularis and the infraspinatus can act as inferior destabilizers in abduction, while in flexion, the subscapularis is the largest potential inferior destabilizer (Ackland and Pandy, 2009; Ackland et al., 2008). We postulated that the ratio of the deltoid muscle compared to the inferior rotator cuff muscle plays a key role in dynamic glenohumeral force balance and, thus, disease progression. In OA patients, less total force, but also lower ratios of stabilizing compressive force to shear force, may potentially lead to bony degeneration due to destabilization. In addition, joint medialization may contribute to rotator cuff muscle shortening and degeneration, with increased joint medialization linked to the increased fatty infiltration of the rotator cuff (Donohue et al., 2018). In contrast, while the RCT patients demonstrate better compression and joint stabilization, they are at a higher risk of tears due to high force magnitudes.

A limitation of our CSA and GI measurements was the selection of manual landmarks by a single clinical expert for each scapula. While these measurements primarily aimed to confirm morphological differences between RCT and OA patient cohorts, there remains uncertainty in landmark positioning, affecting morphological measurements. Future improvements could involve averaging measurements from multiple users for enhanced accuracy, if more precise quantification of the metrics is required. In addition, Suter et al. (2015) conducted a comprehensive study on the impact of the radiographic projection on the CSA. They proposed a method to replicate a true anteroposterior projection of the scapula by adjusting the measurement plane based on glenoid retroversion. In our study, we projected the CSA onto the scapular plane without correcting for version. Suter et al. demonstrated that for retroversion <5°, the difference in the CSA is less than 2°. Our mean retroversion measured 4.4° ± 4.0° for the OA patient group and 2.1° ± 3.1° for the RCT patient group; however, for increased accuracy, the version needs to be considered.

The registration of the generic AnyBody™ scapula to the patient-specific scapula using LDDMM had mean sub-millimeter precision throughout the scapula geometry and showed good conformance at important clinical landmarks (see Table 1). Previous models showed that muscle moment arms are highly sensitive to the attachment point and position relative to the center of motion (Mulla et al., 2019). In our models, the scapular morphing inherently updated the muscle insertion points of the model to match the patient anatomy. Although the moment arms calculated with our models were within the range of values observed during cadaver studies by Ackland et al. (2008), in future work, their accuracy could be verified using measured tendon insertion sites from MRI.

One limitation of our model was the coupling of the glenoid inclination with the position of the glenohumeral joint center. As this has a large influence on the moment arms of the rotator cuff and deltoid (Mulla et al., 2019), we would recommend that the differences in glenohumeral joint position between RCT and OA patients be tested in future work in a model that considers the patient-specific glenohumeral joint center, humerus rotation, and patient posture, for example, obtained using dynamic biplane radiographic imaging. Furthermore, the models in this study used uniformly scaled muscle volumes based on the generic AnyBody™ model, which features muscle recruitment patterns modeled on healthy patients, a generic scapula rhythm to position the scapulothoracic joint (Mulla et al., 2019), and a neutral humerus position (Otis et al., 1994; Ackland et al., 2012), which all have an effect on the moment arms and, thus, the recruitment of the muscles in the model. In future models, it would be helpful to include patient-specific kinematic data (obtained, for example, from dynamic radiographic imaging), patient-specific electromyography (EMG) for muscle activation data, muscle volumetric data (from CT or MRI), and assessments of muscle quality, including fatty infiltration [from CT (Werthel et al., 2022), or MRI(Fuchs et al., 1999)]. Incorporating such data would significantly enhance our ability to comprehensively perform patient modeling. Specifically, the integration of more complex Hill-type muscle models within the biomechanical model would allow for the incorporation of parameters such as physiological cross-sectional area and optimal fiber length, where variations in muscle quality would have a notable impact (Miller et al., 2018). Nikooyan et al. (2010) and Kian et al. (2019) suggested that static optimization in models such as the AnyBody™ shoulder model possibly underestimates antagonistic muscles and, thus, their contribution to joint stability, which could also be addressed by incorporating subject-specific muscle recruitment. Finally, in this model, contact forces and humeral translations were not considered, potentially affecting the balance of forces around the glenoid. Ongoing work to implement humeral translation including ligamentous and cartilaginous stability constraints within the glenohumeral joint (Menze et al., 2023a; Menze et al., 2023b) will enable this to be considered in computational patient-specific modeling of the shoulder in the future.

## 5 Conclusion

This study highlights the importance of considering the complete 3D scapular morphology when studying shoulder biomechanics in specific patient groups. We demonstrated the critical influence of glenohumeral joint center positioning on the moment arms of the rotator cuff and deltoid muscles, with the OA patients having a more medial and inferior glenohumeral joint center than the RCT patients, leading to lower total glenohumeral forces but higher ratios of shear to compression forces. Using our adaptable and customizable pipeline for conducting large-scale, patient-specific biomechanical analyses of the glenohumeral joint, we are poised to make significant strides in elucidating the association between biomechanics and the progression of pathological conditions, paving the way for the development of targeted and personalized treatment strategies by leveraging an individual’s distinct biomechanical profile.

## Data Availability

The data analyzed in this study are subject to licenses/restrictions. The participants whose data were used in this study did not give written consent for their data to be shared publicly. Due to the sensitive nature of the research, supporting data are not published. Requests to access these datasets should be directed to MZ, ses@sonnenhof.ch.
